# An encrypted traffic classification method based on autoencoders and convolutional neural networks

**DOI:** 10.1371/journal.pone.0333276

**Published:** 2025-09-24

**Authors:** Shengwei Xu, Jijie Han, Jianbo Wang, Yijie Bai

**Affiliations:** 1 Information Security Research Institute, Beijing Electronic Science and Technology Institute, Beijing, China; 2 Department of Cryptography Science and Technology, Beijing Electronic Science and Technology Institute, Beijing, China; 3 Department of Cyberspace Security, Beijing Electronic Science and Technology Institute, Beijing, China; Military Institute of Science and Technology, BANGLADESH

## Abstract

To solve the problems of existing encrypted traffic classification methods, such as the need for large-scale training data, high computational costs, and poor generalization ability, an encrypted traffic classification method based on autoencoders and convolutional neural networks was proposed. This method first utilizes an autoencoder to recon-struct the dataset, enabling it to work with smaller-scale datasets. The autoencoder allows shorter traffic flows to learn abstract feature representations from longer traffic flows of the same type, replacing zeros and mitigating the negative effects of zero-padding on traffic classification when using uniform flow lengths. After reconstruction, a convolutional neural network is used to classify the traffic. Due to its characteristics of parameter sharing and local connectivity, the CNN exhibits strong generalization ability when handling tasks, allowing it to better adapt to samples outside of the training data. Experimental results show that, compared to existing advanced methods, this method can achieve a classi-fication accuracy improvement of 2.86% to 18.13%, while also demonstrating greater robustness compared to other advanced methods. The code is available at https://github.com/han20011019/AECCN.

## 1. Introduction

Traffic classification is a critical technology in the field of computer networks. Its primary purpose is to categorize traffic into distinct classes based on specific criteria, enabling network management, cybersecurity assurance, and network structure optimization. This technology is vital for numerous applications, such as quality of service (QoS) control, pricing, resource allocation planning, malware detection, and intrusion detection [1]. Consequently, over the past two decades, both the networking industry and research communities have dedicated significant efforts to studying this technology, resulting in the proposal of many methods and substantial achievements. However, the continuous evolution of the internet and mobile technologies has created a dynamic environment where new applications and services emerge daily, while existing ones undergo constant development [2]. For instance, as nations increasingly prioritize cybersecurity, encrypted communication has become widespread. In such scenarios, performing traffic classification to ensure normal service delivery poses a significant challenge when traffic is fully encrypted. Thus, in this ever-changing network landscape, researching novel traffic classification methods remains essential.

Over time, traffic classification techniques have continued to evolve. The earliest methods were port-based, relying on the assumption that the same protocol or application consistently uses the same port number. However, this simplistic rule-based approach is easily evaded, leading to a steady decline in its accuracy. The next generation of traffic classification technology shifted to deep packet inspection (DPI) [3–5], which analyzes packet payloads to extract specific keywords. Unfortunately, this method is only effective for unencrypted traffic and incurs high computational overhead. Subsequently, researchers [6–10] explored statistical feature extraction combined with classical machine learning (ML) algorithms for encrypted traffic classification. While this approach advanced the field, it heavily relies on expert-designed features, suffers from poor generalization capabilities, and remains susceptible to unreliable flow impacts. Deep learning (DL), in contrast, eliminates the need for domain-specific feature engineering. Through training, DL models automatically learn representative features, capturing nonlinear relationships between raw data and corresponding outputs to form an end-to-end paradigm. This capability has made DL a highly popular method in recent years.

Raw network traffic, at its core, is fundamentally a form of sequential data. Leveraging the ability of recurrent neural networks (RNN) to handle sequential data and accommodate flexible input-output lengths, Kim et al. [11] and Song et al. [12] integrated RNN as foundational components into their classification frameworks. However, RNN suffer from inherent issues such as gradient vanishing and gradient explosion, which hinder their performance on tasks involving long-term dependencies. Long short-term memory (LSTM) networks, a specialized variant of RNN, were designed to address these limitations. Hwang et al. [13] and Thapa et al. [14] adopted LSTM for traffic classification tasks. In a novel approach, Wang et al. [15] transformed 1D sequential traffic data into 2D image-like representations and employed convolutional neural networks (CNN) for learning and classification. While CNN excel at capturing local spatial features through convolutional operations, they require large volumes of labeled training data and exhibit weaker capabilities in modeling global contextual information.

Existing deep learning-based traffic classification methods focus solely on the intrinsic features of traffic flows without considering inter-flow relationships. To address this, several studies [16,17] have attempted to apply graph convolutional networks (GCN) to traffic classification. GCN leverage graph topology to perform learning directly on graph-structured data, aggregating information from neighboring nodes to enhance feature representations. However, in practical classification scenarios, GCN are typically limited to two-layer architectures, primarily because deeper GCN suffer from over-smoothing, where node features become indistinguishable, leading to a sharp decline in accuracy. For large-scale traffic datasets, shallow two-layer GCN are sufficient, as the abundance of data reduces the need to model deep hierarchical relationships. Yet, when applied to small-sample traffic datasets, GCN struggle to achieve satisfactory classification performance due to their limited capacity to uncover intricate patterns in sparse data.

With the rise of large models such as Transformers, many methods based on these models have been proposed. Compared to traditional deep learning approaches, these methods involve a pre-training phase using vast amounts of data, followed by fine-tuning for specific classification tasks to achieve higher accuracy. However, they require extensive data for pre-training, and the pre-training process itself is highly time-consuming.

Although deep learning-based traffic classification methods and Pre-training Models-based methods each have their own strengths and weaknesses, they achieve significantly better performance compared to traditional methods and classical machine learning-based approaches. In recent years, they has gained widespread recognition in academia. However, whether based on ML or DL, most methods adopt flow-level data (e.g., statistical features or raw bytes of traffic) as input, which necessitates uniform input lengths. While various unified lengths have been proposed, zero-padding remains the default technique to align shorter flows to the target length. In small-scale datasets, this introduces substantial noise, as flows of different classes may share long segments of padded zeros, severely degrading classification performance. This consequently leads to their general reliance on large amounts of raw data. The advantages and disadvantages of each method are illustrated in Table 1.

**Table 1:** The advantages and disadvantages of each method.

	Sample size	Computational Complexity	Accuracy
Conventional traffic classification methods	Good	Good	bad
machine learning-based methods	Normal	Good	Normal
Deep learning-based methods	Bad	Normal	Good
Pre-training Models-based methods	Bad	Bad	Good
AECCN	Good	Good	Good

In light of the aforementioned issues, this paper proposes an encrypted traffic classification method based on autoencoder and convolutional neural networks (AECCN). The main contributions of this paper are summarized as follows:

(1) By ingeniously leveraging the properties of autoencoders (AE) [18], we employ AE to reconstruct the dataset. Specifically, AE enable shorter traffic flows to learn abstract feature representations from longer flows of the same class, thereby replacing zero-padding and mitigating its adverse effects during uniform traffic length alignment for classification tasks.
(2) By integrating AE with CNN, we propose a novel encrypted traffic classification method, termed AECCN. Experimental results demonstrate that AECCN effectively addresses the limitation of CNN requiring large-scale raw data for training, achieving high-precision traffic classification even with smaller datasets. Moreover, AECCN employs a lightweight CNN architecture, which effectively reduces its computational complexity. AECCN is an end-to-end encrypted traffic classification model with strong practicality, as it directly accepts raw pcap files as input without requiring specialized preprocessing.
(3) To evaluate the performance of AECCN, we conducted a series of experiments on two public datasets, including accuracy tests and robustness evaluations. The results demonstrate that AECCN achieves a classification accuracy improvement of 2.86%–18.13% compared to state-of-the-art methods, while also exhibiting significantly stronger robustness than existing advanced approaches.

## 2. Related work

With the widespread adoption of encrypted communication technologies, encrypted traffic classification has attracted significant attention. In this section, previous studies are categorized and reviewed based on the methodologies employed.

### 2.1. Conventional traffic classification methods

Conventional traffic classification methods primarily include port-based approaches and DPI. Port-based classification, relying on port numbers, is highly vulnerable to attacks due to the misuse of port information. Subsequently, DPI-based methods emerged. Libprotoident [3], a DPI library, performs application-layer protocol identification by analyzing traffic. Unlike techniques requiring full packet payload capture, Libprotoident uses only the first four bytes of payload in each direction, the size of the first payload-carrying packet per direction, and TCP/UDP port numbers. nDPI [4], employed by ntop and nProbe, enables application-layer protocol detection regardless of port usage—identifying known protocols on non-standard ports and detecting protocols operating against their default ports. OpenDPI [5], derived from Ipoque’s commercial PACE product, integrates techniques like behavioral analysis (searching for application-specific traffic patterns) and statistical profiling (computing metrics to infer transport types). Despite DPI’s success and prevalence in industry products [19,20], recent studies reveal its critical limitation: encrypted traffic blocks access to raw payload data [1], posing significant challenges to DPI’s effectiveness.

### 2.2. Traditional machine learning-based methods

Unlike DPI, traditional machine learning-based methods rely on statistical features, enabling them to handle encrypted traffic. Researchers first design traffic features (e.g., the number of packets, minimum/maximum packet size) based on specific classification requirements (e.g., protocol/traffic type). These features are then fed into different machine learning-based classifiers, including Decision Tree (DT) [21], k-Nearest Neighbors (kNN) [22], and Support Vector Machine (SVM) [23], for classification.

In [10], the authors evaluate the classification performance of multiple machine learning models by utilizing different sets of statistical features. The models employed include DT, kNN, SVM, and Naive Bayes (NB) [24]. The proposed feature sets primarily cover several aspects of the flow, such as ports (protocol flag, source port, and destination port), number of packets, packet size (maximum/minimum/average/ standard of packet size, sizes of the first 10 packets). They conducted experiments using the WEKA machine learning software suite, a well-known machine learning framework with various algorithms built-in, and found that DT exhibited the greatest discriminative capability. More examples include Bernaille et al. [9], who observed that the size and direction of the initial packets of a TCP connection were significant. Based on these features, they proposed a traffic classification model using simple K-Means clustering. Gil et al. [2] utilized kNN and DT algorithms with time-related features, such as the duration of the traffic, the number of bytes per second, and forward/ backward arrival intervals.

These methods break down the whole classification problem into several sub-problems (e.g., feature derivation, machine learning model evaluation) and solve them separately. However, simply combining optimal sub-solutions may not yield a globally optimal solution. Moreover, when faced with different classification requirements, the generalization of manual feature engineering is quite poor [1].

### 2.3. Deep learning-based methods

Compared to traditional machine learning methods, deep learning-based approaches offer two main advantages. Firstly, deep learning methods are end-to-end, meaning they are likely to achieve a globally optimal solution. Secondly, neural networks can learn discriminative features directly from raw inputs, typically the raw bytes of traffic. This automatic learning process involves less manual effort. As network technologies and protocols evolve, some fields in packet headers that were previously unencrypted are now encrypted, such as DOH and TLS 1.3. These changes cause many encrypted classification methods to fail. Leveraging the advantages of automatic feature extraction and end-to-end classification, some studies have attempted to apply deep learning to encrypted traffic classification. Some scholars [25–27] have utilized CNN and LSTM to propagate flow- and packet-based features. Liu et al. [28] used an end-to-end encrypted traffic classification model, which includes an encoder for generating features and a decoder with a reconstruction layer to restore the input sequence. Lotfollahi et al. [29] employed stacked autoencoders and one-dimensional CNN to automatically extract features from encrypted traffic payloads. Sun et al. [16] combined AE with GCN for encrypted traffic classification, unifying the dimensionality of feature representations output by each GCN layer with the AE encoding, and then combining their outputs in a certain proportion as the feature representation learned by each layer of the model.

Deep learning-based methods have achieved significant results compared to conventional methods and traditional machine learning approaches. In recent years, encrypted traffic classification methods based on deep learning have gained widespread recognition in academia. Whether using machine learning or deep learning methods, most approaches employ flow-level data, such as statistical features of traffic or raw bytes, as input. This requires standardizing the input data length. Although the choice of input lengths varies in different studies, zero-padding is usually used to supplement data with insufficient original traffic length. However, when the dataset is small, a large proportion of zero-padding may exist in many traffic samples, which negatively impacts the model’s effective classification, particularly when dealing with multiple types of traffic. Therefore, this paper proposes a novel encrypted traffic classification method (AECCN). AECCN cleverly utilizes the characteristics of AE to reconstruct datasets. Experiments have shown that AE allows shorter traffic to learn abstract feature representations from longer traffic of the same type, thus replacing zeros to mitigate the adverse effects of zero-padding when standardizing traffic length for classification. Moreover, the experiments demonstrated that AECCN effectively overcomes the drawback of CNN requiring a large amount of raw data as a training set and can achieve high-precision traffic classification even with smaller datasets.

### 2.4. Pre-training models-based methods

Transformers excel in parallelization, modeling long-range dependencies, and adaptability across tasks, making them highly efficient and versatile for various applications. To effectively utilize unlabeled data, several traffic classification pretraining models based on Transformers have been proposed. Inspired by BERT’s pretraining method in natural language processing, PERT [30] and ET-BERT [31] tokenize raw traffic bytes, apply masked language modeling to learn traffic representations, and fine-tune the models for downstream tasks. However, Transformer-based models face challenges in computational and memory efficiency due to the quadratic complexity of their core self-attention mechanism.

Mamba is designed for high efficiency, leveraging advanced architecture to optimize feature extraction, reduce computational overhead, and enhance performance across diverse tasks. Wang et al. [32] were the first to apply it to traffic classification, achieving excellent results. Not only does it exhibit very high classification accuracy, but it also outperforms existing methods in terms of computational complexity and efficiency. However, methods based on pre-trained models often require large amounts of raw data, and their performance may degrade in small-sample scenarios.

## 3. Methodology

**Fig 1:**
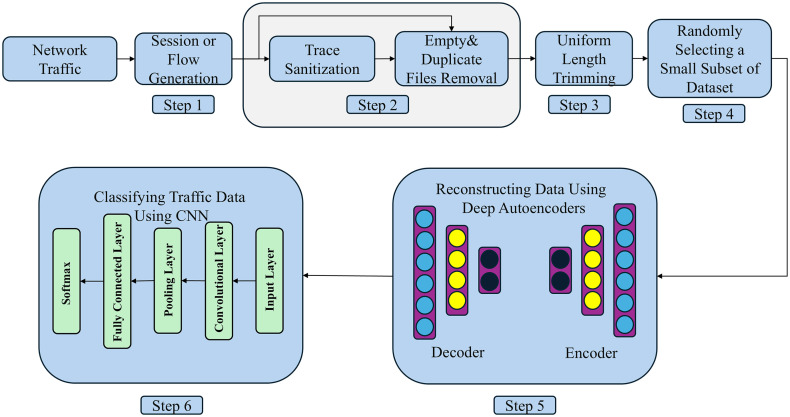
The overall workflow of AECCN.

This section provides a detailed overview of AECCN, with its overall workflow illustrated in Fig 1. The process begins by extracting raw traffic data from the dataset and proceeds through six sequential steps to achieve traffic classification. The first three steps involve preprocessing the raw traffic using USTC-TK2016 [15], a tool developed by Wang et al. for handling pcap files. This preprocessing stage removes redundant or irrelevant data, producing non-repetitive, non-all-zero binary traffic segments standardized to a fixed length of 784 bytes. Following this, the fourth step applies random sampling to generate a representative subset of the preprocessed data for experimental use. The fifth step leverages an AE to reconstruct the sampled data, enhancing feature representation. Finally, the sixth step employs a Convolution CNN to perform the classification task. Subsequent subsections will delve into the technical specifics of each stage to clarify the methodology and its implementation.

### 3.1. Step 1 traffic split

**Fig 2:**
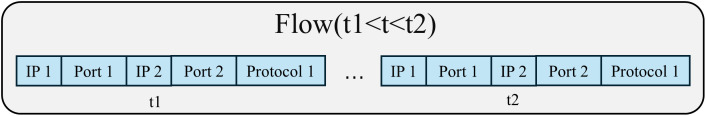
Flow.

**Fig 3:**
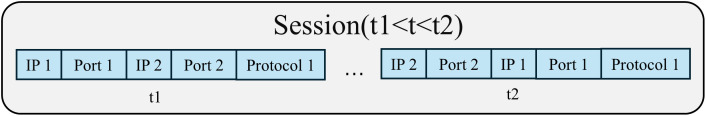
Session.

Split granularity of network traffic includes: TCP connection, flow, session, service, and host [33]. Different split granularity leads to distinct traffic units. The USTC-TK2016 toolkit provides two types of split granularity: flow and session. These two types of split are also widely used in many studies. A flow refers to a group of packets arranged in time order that share the same quintuple (source IP, source port, destination IP, destination port, and transport-level protocol) over a period of time, as shown in Fig 2. A session includes both directions of flows, i.e., the source and destination IP/ port are interchangeable, as shown in Fig 3. Once each packet is grouped according to the specified traffic split granularity, USTC-TK2016 provides two processing options for each packet itself: L7 and ALL. L7 refers to retaining only layer 7 of the OSI model, while ALL refers to retaining all layers. In this paper, all experimental data are processed using the Session+L7 method.

### 3.2. Step 2 traffic clear

In this step, the process begins with trace sanitization, which involves randomizing the MAC address at the data link layer and the IP address at the IP layer, respectively. This is optional, for example, when all traffic is from the same network, the MAC and IP may no longer be the distinguishing information, and we don’t need to perform it in this situation. In this paper, trace sanitization is not required because only application layer data is retained for each data packet. Next, the traffic files are cleaned, primarily by deleting empty files and duplicate files.

### 3.3. Step 3 uniform length trimming

After processing the data in the first two steps, we have obtained valid traffic data rather than discrete data packets from a real network environment. However, these data cannot be used for deep learning because they vary in length, so all traffic data must be transformed to a uniform length. Wang et al. [15] trimmed all data to 784 bytes, Sun et al. [16] trimmed all data to 900 bytes, and Xie et al. [34] experimented with trimming data to 40, 50, and 60 bytes respectively. In this paper, we trimmed all data to 784 bytes. For traffic longer than 784 bytes, the first 784 bytes are taken; for traffic shorter than 784 bytes, it is padded with zeros up to 784 bytes.

### 3.4. Step 4 random sampling

In recent years, researchers have achieved significant progress in traffic classification, as discussed in Section II of this article. This success is largely attributed to the availability of large-scale publicly available traffic datasets, where models can attain robust performance through extended training periods. AECCN, however, overcomes the limitation of traditional CNN that require massive raw data for training. Consequently, this study only needs to extract a small subset of data from these public datasets for experimentation. Specifically, for all datasets used in this work, 200 samples per traffic class are randomly selected to ensure efficient and representative training while maintaining methodological rigor.

### 3.5. Step 5 reconstruction of the dataset

**Fig 4:**
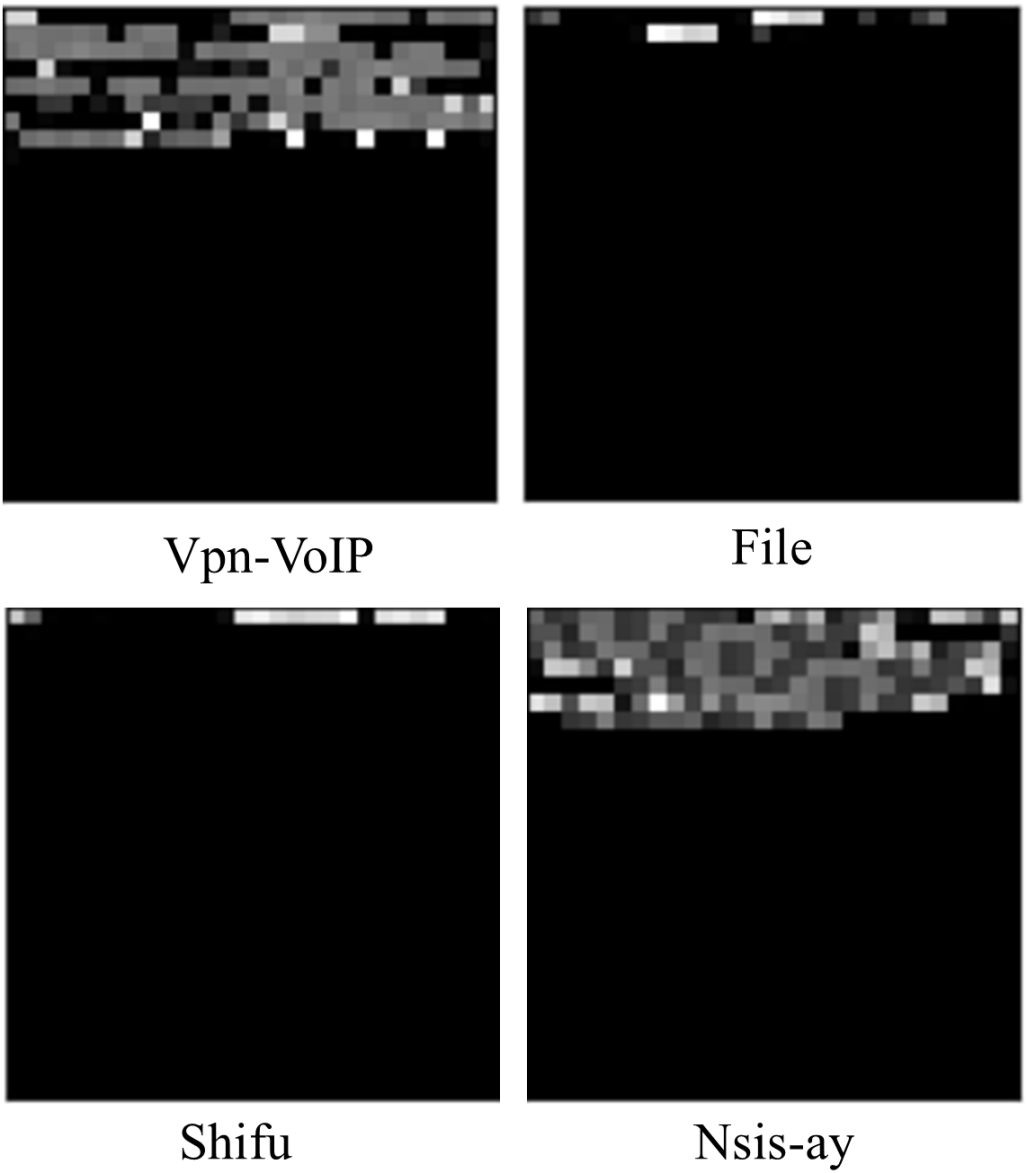
Part of the traffic after zero-padding.

In the third step, all traffic data underwent a process of length standardization, where data with a length less than 784 bytes was padded with zeros. Although this approach is simple and efficient, it is not conducive to subsequent deep learning classification. This indiscriminate operation can lead to a long section of identical data (all zeros) at the end of different types of traffic data, which is detrimental to classification. Additionally, some traffic data naturally have very small lengths, such as packets that send control instructions. As illustrated in Fig 4, for ease of visualization, each byte of traffic data is converted into an integer value between 0 and 255, and then the 784-byte data is transformed into a 28*28 matrix of integers. These integers are then converted to grayscale values to display the matrix as an image, where the black areas represent values of 0. These packets with very short lengths appear across different categories of data, and padding them with zeros to 784 bytes complicates the classification process further.

**Fig 5:**
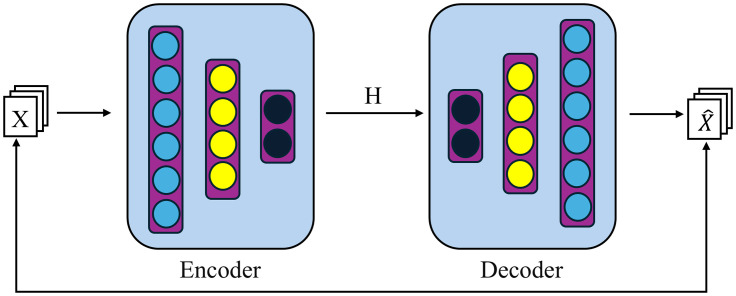
Autoencoder.

To address the issues mentioned above, this paper proposes a data reconstruction method using AE [18] to represent the data. The basic principle of the AE is illustrated in Fig 5. An AE comprises two parts: an encoder and a decoder. The encoder compresses the original data 𝐗 into 𝐇 as an abstract feature representation for 𝐗. The decoder uses this feature vector 𝐇 to generate reconstructed data $\hat{X}$. The loss function measures the difference between the original data 𝐗 and the reconstructed data $\hat{X}$.

When using AE to reconstruct traffic data, the encoder’s dimensions were set to 784-128-64-32, and the decoder’s dimensions were set to 32-64-128-784. The original dimension of the traffic data is 784, while the hidden layers in the encoder contain 128 and 64 neurons, respectively. The learned feature representation dimension is 32. During training, the model ran for 10 epochs, using the Adam optimizer and the binary cross-entropy loss function.

Feature vector 𝐇 can be generated by Eq (1):


$${𝐇}_{e}^{\left(m\right)}=\sigma ({𝐖}_{e}^{\left(m\right)}{𝐇}_{e}^{\left(m-1\right)}+{𝐛}_{e}^{\left(m\right)})$$ (1)

Assuming the encoder consists M layers, m in Eq (1) denotes the m -th layer of the encoder, where 1≤m≤M. e means it is the variable in the encoder. ${𝐇}_{e}^{\left(m\right)}$ denotes the feature representation learned through the m -th layer of the encoder. ${𝐖}_{e}^{\left(m\right)}$ and ${𝐛}_{e}^{\left(m\right)}$ denote the weight matrix and biases in the m -th layer of the encoder, respectively. σ denotes the activation function of the full connection layer such as ReLU [35] or Sigmoid function. Additionally, we define ${𝐇}_{e}^{0}$ as the original data 𝐗 and $H_{e}^{\left(M\right)}$ as the feature vector 𝐇.

The reconstructed data $\hat{X}$ can be obtained from Eq (2).


$${𝐇}_{d}^{\left(m\right)}=\sigma ({𝐖}_{d}^{\left(m\right)}{𝐇}_{d}^{\left(m-1\right)}+{𝐛}_{d}^{\left(m\right)})$$ (2)

In the Eq (2), m denotes the m -th layer of the decoder, where 1≤m≤M, d means it is the variable in the decoder. ${𝐇}_{d}^{\left(m\right)}$ refers to the reconstructed data at the m -th layer of the decoder. ${𝐖}_{d}^{\left(m\right)}$ and ${𝐛}_{d}^{\left(m\right)}$ denote the weight matrix and biases of the m -th layer of the decoder, respectively. Additionally, we define ${𝐇}_{d}^{0}$ as the feature vector 𝐇 and ${𝐇}_{d}^{\left(M\right)}$ as the reconstructed data $\hat{X}$.

The loss function $L_{R}$ can be derived from Eq (3).


$$L_{R}=\frac{\text{1}}{2N}\sum_{i=1}^{N}{\left\|{𝐱}_{i}-\;\hat{{𝐱}_{i}}\right\|}_{2}^{2}=\frac{\text{1}}{2N}||𝐗-\hat{𝐗}|{|}_{F}^{2}$$ (3)

In Eq (3) , N represents the quantity of reconstructed data in the training set.

In this paper, we convert each traffic into integers ranging from 0 to 255, essentially transforming single traffic into 784 integers to accommodate AE for computation. 𝐗 represents each traffic. We reconstruct each type of traffic data in the dataset separately, use 80% of the traffic to train AE, and then reconstruct the remaining 20% of the traffic. Specifically, we use 160 items of the traffic from each type as the training set, then use AE to reconstruct the remaining 40 items. Consequently, each type in the dataset contains only 40 items in subsequent work.

**Fig 6:**
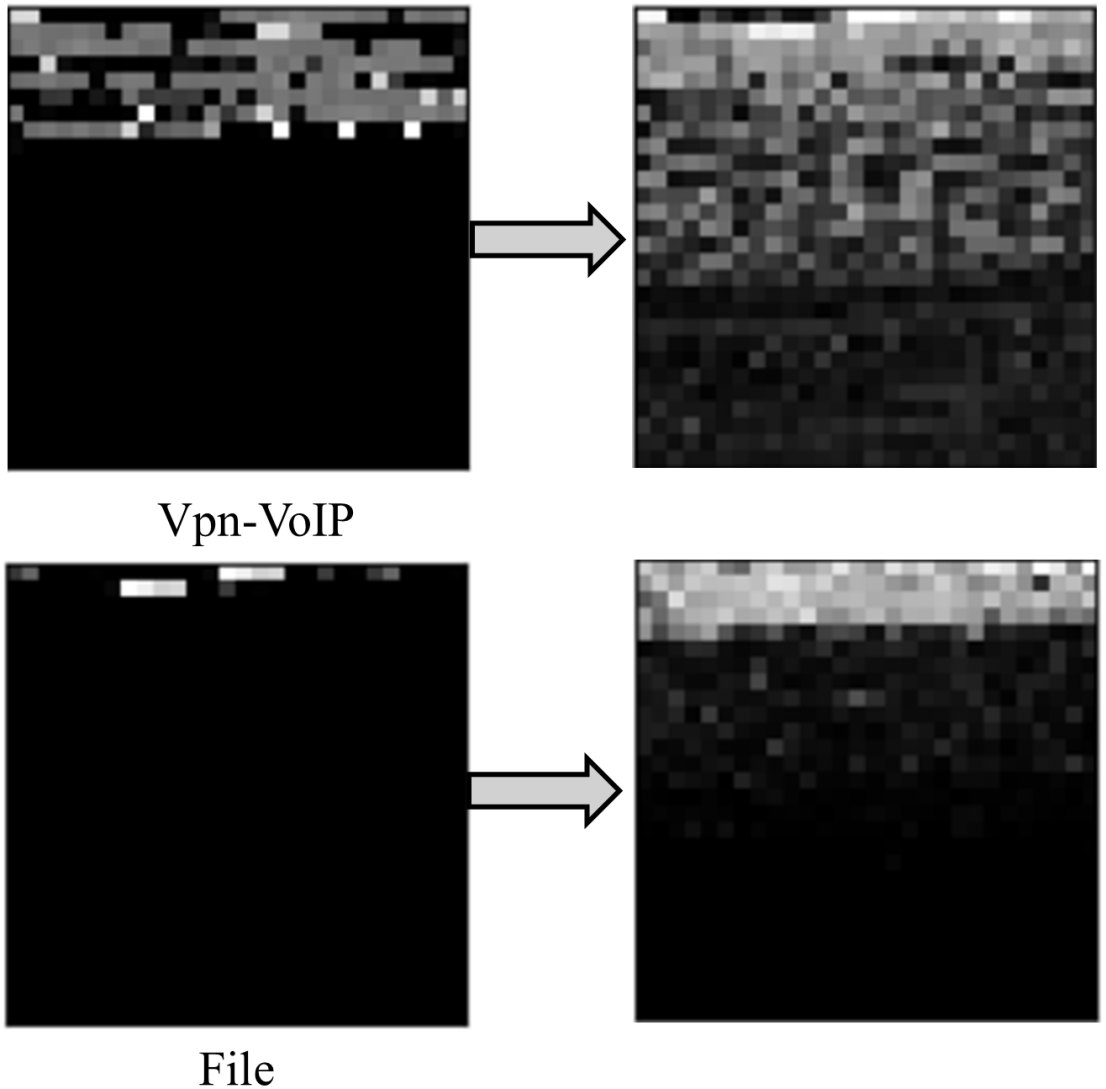
The comparison between traffic reconstructed by AE and zero-padding.

**Fig 7:**
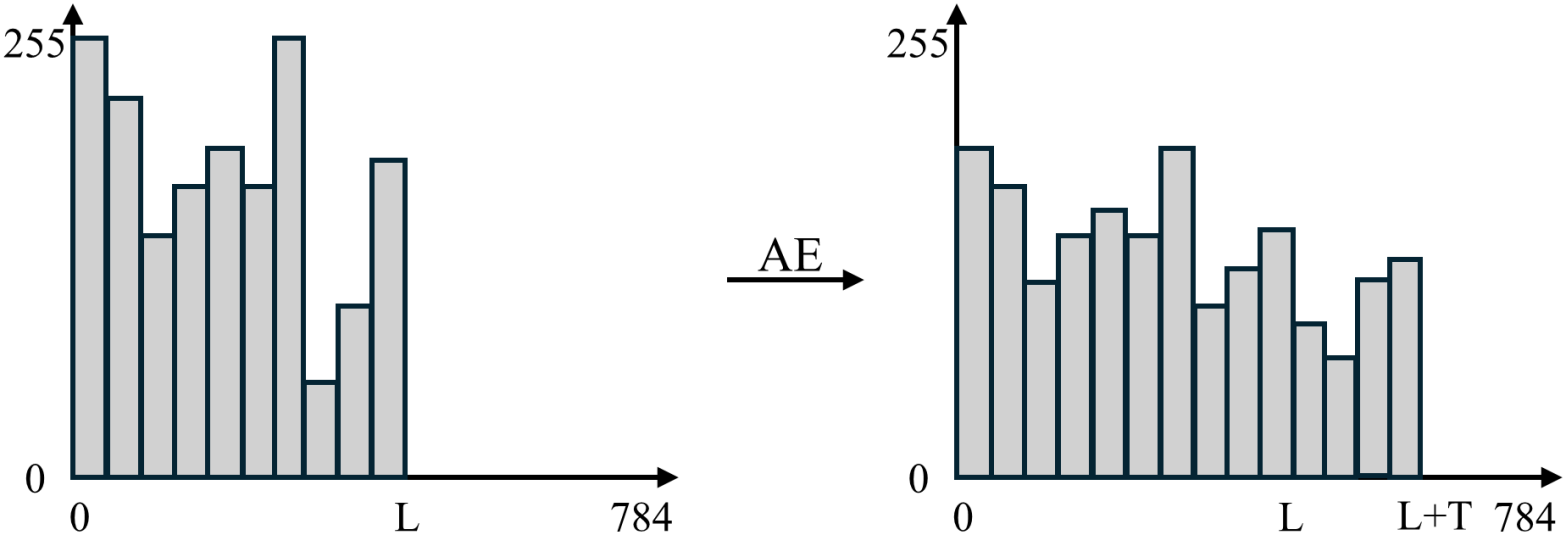
The abstract representation of reconstructing traffic by AE.

The effect of reconstructing data using AE is shown in Fig 6. This reconstruction effect can be abstractly represented by Fig 7. Under the experimental setup described in this paper, an AE reconstruction takes approximately 0.1 seconds.

To quantitatively analyze the changes brought by using AE for traffic reconstruction, we employed Principal Component Analysis (PCA) to calculate four key metrics for both original and reconstructed traffic: dimensions required to retain 95% data information, mean intra-class variance, mean inter-class variance, separation index. The results are presented in Table 2. These findings clearly demonstrate that the primary function of AE is to reduce intra-class distances – effectively transforming originally dispersed traffic samples of the same class into more compact clusters. This observation aligns perfectly with our theoretical predictions.

**Table 2:** Quantitative metrics comparison for traffic data reconstruction using AE.

		dimensions(95%)	intra-class	inter-class	separation index
Iscx	original	317	36.01	23.97	0.67
	AE-reconstructed	11	15.49	24.88	1.61
Vpn	original	247	39.77	24.49	0.69
	AE-reconstructed	8	16.16	29.22	1.81
novpn	original	192	32.76	20.13	0.61
	AE-reconstructed	6	14.83	20.33	1.37
Ustc	original	449	37.21	48.47	1.30
	AE-reconstructed	11	11.74	48.34	4.12
malware	original	330	23.91	22.06	0.92
	AE-reconstructed	7	7.65	22.12	2.89
normal	original	375	50.79	71.04	1.40
	AE-reconstructed	7	15.83	69.62	4.40

AE can appropriately extend traffic that was only of length L before zero-padding to a length of (L+T), and can also reduce the variance of individual bit values in the original data. This results in a smoother variation in the values of individual bits within the traffic data. Specifically, during the feature extraction phase, the encoder maps both long and short flows from the same traffic category into a shared latent space, where their high-level features (e.g., protocol patterns, payload structures) are encoded as dense vectors. Throughout the training process, the AE optimizes the latent representations to align short flows with their longer counterparts within the same class. This approach is grounded in the fundamental assumption that flows from the same category share discriminative characteristics. In other words, for short flow samples, the decoder leverages the loss function (such as mean squared error) to naturally drive the latent representations of short flows to approximate the distributional features of longer flows, enabling reconstruction without requiring explicit interpolation.

### 3.6. Step 6 traffic classification

**Fig 8:**
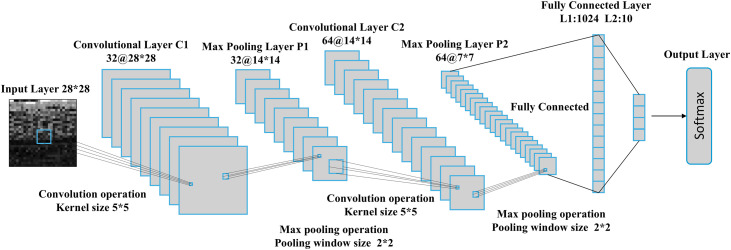
The basic process of using CNN for traffic classification.

Following Step 5, 40 reconstructed traffic samples are obtained for each traffic class. The final classification is performed using a CNN, with the core workflow illustrated in Fig 8. The CNN first reads 28 × 28 × 1 grayscale traffic images from files, normalizing pixel values from the [0, 255] range to [0, 1]. The initial convolutional layer C1 applies 32 convolutional kernels of size 5 × 5, generating 32 feature maps of size 28 × 28. A 2 × 2 max-pooling operation is then applied to the output of C1, resulting in 32 pooled feature maps of size 14 × 14 (layer P1). The second convolutional layer C2 employs 64 kernels of size 5 × 5, producing 64 feature maps of size 14 × 14. These are further reduced to 64 feature maps of size 7 × 7 via another 2 × 2 max-pooling layer (P2). The pooled features are flattened and passed through two fully connected layers with dimensions 1024 and 10, respectively. The output layer utilizes a Softmax function to compute class probabilities, while dropout is integrated to mitigate overfitting during training.

This architecture balances feature extraction and generalization, enabling robust traffic classification with limited data. In this paper, the CNN architecture is specifically designed for a low-resolution (28 × 28 grayscale) and relatively simple classification task. Given the straightforward nature of the task, the network adopts a shallow structure with only two convolutional layers, each employing 5 × 5 kernels to effectively capture essential features while maintaining computational efficiency. The 2 × 2 max-pooling layers are incorporated following each convolutional layer, a widely adopted strategy in classification tasks to progressively reduce spatial dimensions while preserving dominant features. For the fully connected layers, a two-layer configuration is implemented to align with the lightweight requirements of the task, ensuring a balance between model capacity and computational overhead. This design choice reflects a deliberate trade-off between performance and efficiency for low-complexity image classification. In Table 3, we compare the parameter count and FLOPs of AECCN against mainstream ResNet networks. The results demonstrate that AECCN is significantly lighter than conventional CNNs.

**Table 3:** Comparative analysis: AECCN against mainstream ResNet architectures.

	ResNet-18-layer	34-layer	50-layer	101-layer	152-layer	AECCN
FLOPs	1.8*10^9^	3.6*10^9^	3.8*10^9^	7.6*10^9^	11.3*10^9^	2.8*10 ^**7**^
Parameter counts	11.7*10^6^	21.8*10^6^	25.6*10^6^	44.5*10^6^	60.2*10^6^	0.67*10 ^**6**^

## 4. Experiments

Section 4.1 outlines the foundational preparations for the experiments, including the datasets, experimental environment, and evaluation metrics. Section 4.2 compares AECCN against five alternative approaches, spanning both traditional machine learning and deep learning methods, to demonstrate its competitive advantages. Section 4.3 further evaluates the robustness of AECCN through rigorous testing under extreme conditions, highlighting its resilience and stability in challenging scenarios. Together, these sections systematically validate the methodology’s effectiveness, generalizability, and practical applicability. Section 4.4 to enhance the interpretability of the proposed method, ablation experiments were conducted on AECCN.

### 4.1. Experiment setup

#### 4.1.1. Datasets.

To verify the reliability of AECCN and enhance the credibility of the experimental results, all experiments in this paper are conducted using two public traffic datasets: USTC-TFC2016 and ISCX-VPN-NonVPN-2016. These datasets were both collected from real network environments and consist of raw traffic data. Detailed introductions to the two datasets are as follows:

USTC-TFC2016

The dataset was established by Wang et al. [15] and consists of two parts, as shown in Tables 4 and 5. The first part includes malware traffic from 10 real network environments, obtained by CTU researchers from public websites between 2011 and 2015 [36]. In some cases, a portion of larger-scale traffic was used, while smaller-scale traffic was merged with similar types. The second part comprises normal traffic from 10 real network environments, collected by the creators using IXIA BPS [37].

**Table 4:** USTC-TFC2016 Part I (Malware Traffic).

Traffic Type	CTU num	Binary MD5	process
Cridex	108−1	25b8631afeea279ac00b2da70fffe18a	original
Geodo	119−2	306573e52008779a0801a25fafb18101	part
Htbot	110−1	e515267ba19417974a63b51e4f7dd9e9	original
Miuref	127−1	a41d395286deb113e17bd3f4b69ec182	original
Neris	42,43	bf08e6b02e00d2bc6dd493e93e69872f	merged
Nsis-ay	53	eaf85db9898d3c9101fd5fcfa4ac80e4	original
Shifu	142−1	b9bc3f1b2aace824482c10ffa422f78b	part
Tinba	150−1	e9718e38e35ca31c6bc0281cb4ecfae8	part
Virut	54	85f9a5247afbe51e64794193f1dd72eb	original
Zeus	116−2	8df6603d7cbc2fd5862b14377582d46	original

**Table 5:** USTC-TFC2016 Part II (Normal Traffic).

Traffic Type	Class	Traffic Type	Class
BitTorrent	P2P	Outlook	Email/WebMail
Facetime	Voice/Video	Skype	Chat/IM
FTP	Data Transfer	SMB	Data Transfer
Gmail	Email/WebMail	Weibo	Social NetWork
MySQL	Database	WorldOfWarcraft	Game

ISCX-VPN-NonVPN-2016

The dataset was collected by Draper-Gil et al. [2] using Wireshark and tcpdump from a real network environment, where laboratory members created accounts and used services like Skype and Facebook, as shown in Table 6. The dataset comprises 7 categories of data, each with normal and VPN protocol-encapsulated data formats, leading to a total of 14 labels. However, since Wang et al. [15] and Xie et al. [34] both noted issues with the “Browser” and “VPN-Browser” data in the dataset, our experiments only used the remaining portion of the dataset, amounting to a total of 12 labels.

**Table 6:** ISCX-VPN-NonVPN-2016.

Traffic Type	Content
Browser	Firefox and Chrome
VPN-Browser	
Email	SMPTS, POP3S and IMAPS
VPN-Email	
Chat	ICQ, AIM, Skype, Facebook and Hangouts
VPN-Chat	
Streaming	Vimeo and Youtube
VPN-Streaming	
File transfer	Skype, FTPS and SFTP using Filezilla and an external service
VPN-File transfer	
VoIP	Facebook, Skype and Hangouts voice calls (1h duration)
VPN-VoIP	
P2P	uTorrent and Transmission (Bittorrent)
VPN-P2P	

#### 4.1.2. Experimental environment.

All experiments were conducted on a laptop equipped with an Intel(R) Core(TM) i5-9300H @ 2.40GHz CPU, 16.0 GB RAM, GTX 1650 GPU, and Windows 11 Home Edition OS. We used PyTorch as the deep learning software framework to implement our methods, with version 2.1.0 and Python version 3.11.5.

#### 4.1.3. Evaluation indexes.

To compare the classification performance of AECCN with other methods, we used four popular indexes: accuracy, precision, recall, and F1 score.

Accuracy can be obtained by Eq (4).


$$Accuracy\mathrm{\backslash nolimits}=\frac{TP+TN}{TP+FP+FN+TN}$$ (4)

Precision can be obtained by Eq (5).


$$Precision\mathrm{\backslash nolimits}=\frac{TP}{TP+FP}$$ (5)

Recall can be obtained by Eq (6).


$$Recall\mathrm{\backslash nolimits}=\frac{TP}{TP+FN}$$ (6)

F1score can be obtained by Eq (7).


$$F\mathrm{\backslash nolimits}1-Score=\frac{2Precision\cdot Recall}{Precision+Recall}$$ (7)

In the aforementioned equation, TP refers to the number of instances correctly classified as a particular category. FP refers to the number of instances incorrectly classified as that category. FN refers to the number of instances that should have been classified as a particular category but were classified as other categories. TN refers to the number of instances correctly classified as not being a particular category.

### 4.2. Contrast experiment

In this section, to validate the effectiveness of AECCN, we compare it with six other methods: KNN [26], GCNII [38], CNN [15], SAM [34], ET-BERT [31], and NetMamba [32]. KNN is a traditional machine learning-based method, while GCNII, CNN, and SAM are deep learning-based methods. ET-BERT and NetMamba is a pre-learning-based method. For KNN, we set K to 5. For GCNII, the number of layers is set to 2, α is set to 0.1, λ is set to 0.5, the hidden layer dimension is set to 64, the learning rate is set to 0.005, and dropout is set to 0.6. For CNN, the number of convolutional layers is 1, the kernel size is 3×3, the input channel size is 1, the output channel size is 32, the number of fully connected layers is 1, and the learning rate is 0.005. For SAM, L is set to 50, and other parameters are kept at their defaults. For ET-BERT, batch_size is set to 16, seq_length is set to 32, and other parameters are kept at their defaults. For NetMamba, epochs for pre-training is set to 400, and other parameters are kept at their defaults.

In AECCN, when using AE for data reconstruction, each class uses 160 traffic samples as the training set, and the trained AE is then used to reconstruct the remaining 40 traffic samples. When using CNN for traffic classification, the training set accounts for 80% and the testing set for 20%. For KNN, 80% of the samples are used for training and 20% for testing. For CNN, the training set accounts for 80% and the testing set for 20%. In GCNII, the training set accounts for 50%, the validation set for 25% and the testing set for 25%. In SAM, the training set accounts for 50% and the testing set for 50%. In NetMamba and ET-BERT, the training set accounts for 80%, the validation set for 10% and the testing set for 10%. All experiments are conducted on datasets containing 200 samples per class. The reported experimental results are the average of 20 experiments and The confidence level is 0.95. All experiments were conducted using 5-fold cross-validation to ensure robust and reproducible results.

The experimental results shown in Table 7 are obtained from 20-class classification on the USTC-TFC2016 dataset. AECCN demonstrates a 13.62% improvement in accuracy compared to SAM, which performed relatively well.

**Table 7:** Results of all methods on the USTC-TFC2016 dataset for 20-class classification.

	Accuracy	Precision	Recall	F1-Score
KNN	63.95 ± 0.16	81.52 ± 0.26	63.95 ± 0.16	71.67 ± 0.14
CNN	79.53 ± 0.17	79.69 ± 0.15	80.1 ± 0.15	79.9 ± 0.11
GCNII	47.63 ± 0.2	67.17 ± 0.22	55.31 ± 0.17	47.02 ± 0.19
SAM	84.39 ± 0.11	85.63 ± 0.11	83.99 ± 0.11	84.8 ± 0.09
NetMamb	81.57 ± 0.13	81.42 ± 0.33	81.57 ± 0.13	81.49 ± 0.16
ET-BERT	82.02 ± 0.13	83.35 ± 0.14	82.02 ± 0.13	82.68 ± 0.11
AECCN	98.01 ± 0.1	98.52 ± 0.12	98.65 ± 0.07	98.78 ± 0.09

The experimental results shown in Table 8 are obtained from 10-class classification on the malicious traffic portion of the USTC-TFC2016 dataset. AECCN demonstrates a 8.69% improvement in accuracy compared to SAM, which performed relatively well. The data reveals that AECCN achieved an accuracy rate of 100%. Table 2 lists the intra-class distance and inter-class s distance for each dataset. It can be observed that the intra-class distance for the malware dataset is only 7.65, which is also the smallest among all dataset categories. This further validates the credibility of our experimental results.

**Table 8:** Results of all methods on the USTC-TFC2016-MALWARE dataset for 10-class classification.

	Accuracy	Precision	Recall	F1-Score
KNN	71.42 ± 0.18	80.49 ± 0.18	71.42 ± 0.18	75.68 ± 0.12
CNN	88.67 ± 0.14	88.87 ± 0.1	89.25 ± 0.16	89.06 ± 0.1
GCNII	52.99 ± 0.17	69.59 ± 0.22	60.45 ± 0.13	53.43 ± 0.2
SAM	91.31 ± 0.14	92.1 ± 0.16	91.42 ± 0.13	91.76 ± 0.11
NetMamb	81.05 ± 0.13	77.25 ± 0.12	81.05 ± 0.13	79.11 ± 0.09
ET-BERT	74.04 ± 0.13	78.1 ± 0.1	74.04 ± 0.13	76.01 ± 0.08
AECCN	100	100	100	100

The experimental results presented in Table 9 are derived from 10-class classification on the normal traffic portion of the USTC-TFC2016 dataset. AECCN demonstrates a 8.2% improvement in accuracy compared to ET-BERT, which performed relatively well.

**Table 9:** Results of all methods on the USTC-TFC2016-NORMAL dataset for 10-class classification.

	Accuracy	Precision	Recall	F1-Score
KNN	65.68 ± 0.18	79.7 ± 0.21	65.68 ± 0.18	72.02 ± 0.13
CNN	81.94 ± 0.14	80.92 ± 0.16	80.6 ± 0.13	80.76 ± 0.12
GCNII	56.9 ± 0.2	80.01 ± 0.19	63.95 ± 0.15	53.26 ± 0.21
SAM	85.5 ± 0.17	85.53 ± 0.14	85.13 ± 0.13	85.33 ± 0.09
NetMamb	81.37 ± 0.14	82.75 ± 0.14	81.37 ± 0.14	82.05 ± 0.08
ET-BERT	90.53 ± 0.13	92.79 ± 0.14	90.53 ± 0.13	91.77 ± 0.12
AECCN	98.73 ± 0.08	98.79 ± 0.09	98.13 ± 0.07	97.48 ± 0.1

The experimental results presented in Table 10 are derived from 12-class classification on the ISCX-VPN-NonVPN-2016 dataset. AECCN demonstrates a 18.13% improvement in accuracy compared to CNN, which performed relatively well.

**Table 10:** Results of all methods on the ISCX-VPN-NonVPN-2016 dataset for 12-class classification.

	Accuracy	Precision	Recall	F1-Score
KNN	54.78 ± 0.19	61.93 ± 0.11	54.78 ± 0.19	58.13 ± 0.12
CNN	70.38 ± 0.11	75.39 ± 0.16	69.37 ± 0.15	72.26 ± 0.13
GCNII	56.61 ± 0.18	63.74 ± 0.22	60.74 ± 0.15	58.02 ± 0.22
SAM	59.95 ± 0.11	64.36 ± 0.1	64.52 ± 0.12	64.44 ± 0.07
NetMamb	66.15 ± 0.14	69.17 ± 0.14	66.15 ± 0.14	67.62 ± 0.09
ET-BERT	54.59 ± 0.13	54.23 ± 0.14	54.59 ± 0.13	54.41 ± 0.1
AECCN	88.51 ± 0.12	92.4 ± 0.1	93.24 ± 0.42	94.5 ± 0.1

The experimental results shown in Table 11 are obtained from 6-class classification on the ISCX-VPN-NonVPN-2016 dataset, focusing on traffic encapsulated through VPN protocols. AECCN demonstrates a 2.86% improvement in accuracy compared to NetMamb, which performed relatively well.

**Table 11:** Results of all methods on the ISCX-VPN-NonVPN-2016-VPN dataset for 6-class classification.

	Accuracy	Precision	Recall	F1-Score
KNN	82.67 ± 0.21	84.91 ± 0.22	82.67 ± 0.21	83.77 ± 0.12
CNN	92.83 ± 0.15	93.11 ± 0.16	93.01 ± 0.15	93.06 ± 0.1
GCNII	86.78 ± 0.18	87.63 ± 0.24	86.99 ± 0.15	86.38 ± 0.23
SAM	90.19 ± 0.16	89.99 ± 0.16	89.82 ± 0.14	89.83 ± 0.11
NetMamb	95.08 ± 0.16	98.66 ± 0.12	95.08 ± 0.16	96.84 ± 0.1
ET-BERT	84.07 ± 0.19	85.24 ± 0.14	84.07 ± 0.19	84.65 ± 0.12
AECCN	97.94 ± 0.11	98.66 ± 0.12	98.56 ± 0.08	98.48 ± 0.11

The experimental results shown in Table 12 are obtained from 6-class classification on the ISCX-VPN-NonVPN-2016 dataset, focusing on regular encrypted traffic. AECCN demonstrates a 17.44% improvement in accuracy compared to CNN, which performed relatively well.

**Table 12:** Results of all methods on the ISCX-VPN-NonVPN-2016-NoVPN dataset for 6-class classification.

	Accuracy	Precision	Recall	F1-Score
KNN	55.47 ± 0.17	59.94 ± 0.18	55.47 ± 0.17	57.62 ± 0.1
CNN	72.16 ± 0.19	82.19 ± 0.12	72 ± 0.16	76.76 ± 0.1
GCNII	56.88 ± 0.17	58.42 ± 0.2	57.38 ± 0.16	56.36 ± 0.18
SAM	57.55 ± 0.14	60.65 ± 0.1	59.63 ± 0.13	60.13 ± 0.09
NetMamb	62.43 ± 0.11	65.25 ± 0.15	62.43 ± 0.11	63.73 ± 0.09
ET-BERT	50.91 ± 0.14	51.81 ± 0.14	50.91 ± 0.14	51.36 ± 0.14
AECCN	89.6 ± 0.1	92.41 ± 0.11	92.08 ± 0.07	91.75 ± 0.09

**Fig 9:**
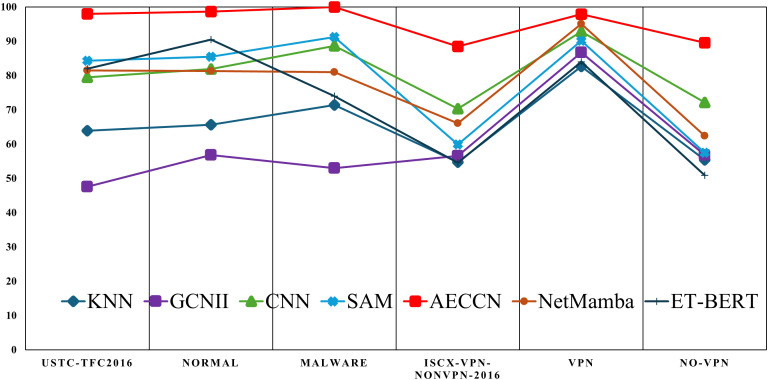
The accuracy comparison of the seven methods across different traffic classification scenarios. The vertical axis represents accuracy measured in percentage (%), while the horizontal axis denotes various traffic classification scenarios.

As shown in Fig 9, we compare the accuracy of these methods across the six scenarios of traffic classification. We observe that AECCN consistently maintains the highest accuracy across all six scenarios, with relatively stable results. The accuracy of the other six methods fluctuates significantly across different classification scenarios, and none of them consistently outperforms the others.

This clearly demonstrates that AECCN is suitable for various traffic classification scenarios. In addition, it maintains high accuracy in the two most challenging scenarios (12-class classification on the ISCX-VPN-NonVPN-2016 dataset and 6-class classification on normal encrypted traffic in the ISCX-VPN-NonVPN-2016 dataset), with accuracies of 88.51% and 89.6%, respectively. This represents an improvement of 18.13% and 17.44% over the other six methods, respectively. Furthermore, pre-trained large models have been widely adopted across various domains in recent years. The mentioned approaches in this paper—NetMamba, ET-BERT, and the recent TranSync—all demonstrate exceptional performance, consistently achieving classification accuracies exceeding 95%. Nevertheless, such large models fail to maintain effectiveness when applied to limited datasets.

From a method-centric perspective, we analyze the performance of each classification method across different scenarios. Notably, all methods exhibit inferior classification performance in two specific scenarios: the 12-class classification on the ISCX-VPN-NonVPN-2016 dataset and the 6-class classification on normal encrypted traffic from the same dataset. To investigate this phenomenon, we examine the actual effective length of traffic flows in different classification scenarios. As presented in Tables 13 and 14, we measured the effective length of all traffic categories in both datasets by calculating the actual data length after trimming to 784 bytes and excluding zero-padding, with all values representing averaged results. This analysis reveals that the performance degradation in these two scenarios may be attributed to their shorter effective traffic lengths compared to other scenarios, providing important insights into the challenges faced by traffic classification methods.

**Table 13:** The average length of each traffic category in the USTC-TFC2016 dataset.

Malware(536.96)		Normal(567.26)	
Type	Length	Type	Length
BitTorrent	603.8	Cridex	784
Facetime	252.79	Geodo	614.15
FTP	167.32	Htbot	533.92
Gmail	728.07	Miuref	768.01
MySQL	113.92	Neris	582.5
Outlook	778.87	Nsis-ay	569.87
Skype	561.6	Shifu	162.35
SMB	654.47	Tinba	160.84
Weibo	783.98	Virut	735.74
WorldOfWarcraft	451.74	Zeus	761.2

**Table 14:** The average length of each traffic category in the ISCX-VPN-NonVPN-2016 dataset.

VPN(461.98)		NO-VPN(292.14)	
Type	Length	Type	Length
VPN-Email	597.85	Email	152.18
VPN-Chat	311.73	Chat	92.61
VPN-Streaming	495.12	Streaming	574.35
VPN-File transfer	498.71	File transfer	475.74
VPN-VoIP	135.52	VoIP	159.56
VPN-P2P	732.98	P2P	298.41

Our analysis reveals a clear correlation between traffic flow length and classification performance across different scenarios. The two scenarios exhibiting the weakest classification results – the 12-class and 6-class classifications on the ISCX-VPN-NonVPN-2016 dataset – consistently show shorter average traffic lengths compared to other scenarios. This observation provides empirical support for our AE-based approach, as the reconstruction mechanism allows shorter flows to effectively learn feature representations from longer flows within the same category, thereby overcoming the limitations imposed by zero-padding. This explains why AECCN achieves superior performance in these challenging scenarios compared to conventional methods. However, the experimental results also highlight an inherent limitation of our approach: even with AE enhancement, AECCN’s performance in short-flow-dominated scenarios remains inferior to its performance in scenarios with longer average flow lengths. This performance bottleneck suggests that while our method significantly improves classification of short flows, there remains room for further optimization when dealing with traffic environments where short flows predominate. These findings not only validate the effectiveness of our proposed solution but also identify important directions for future research in traffic classification.

These findings not only validate the effectiveness of AECCN but also identify important directions for future research in encrypted traffic classification. Recent breakthroughs in other domains suggest promising pathways to address these limitations:

Recent AI advances offer valuable insights for encrypted traffic classification. SpectralGPT’s [39] hierarchical transformer demonstrates strong sequential pattern learning, while cross-city [40] learning methods provide robust domain adaptation frameworks. LRR-Net’s [41] lightweight recurrent architecture shows promise for efficient traffic analysis. These approaches suggest three key directions: foundation models for pretraining, domain adaptation techniques, and efficient sequential architectures – all potentially beneficial for encrypted traffic classification.

### 4.3. Robustness experiment

To demonstrate the stability of AECCN, in this section, we conducted robustness experiments on AECCN, KNN, CNN, SAM, NetMamba and ET-BERT to compare their performance under stringent conditions. As GCNII has lower accuracy, we did not include it in the robustness experiments in this section. In Section 4.3.1, we conducted experiments on dataset sample quantity robustness. In Section 4.3.2, we conducted experiments on the robustness of original data noise levels.

#### 4.3.1. Robustness of datasets size.

In practical applications, it is possible that the existing data may not meet the requirement of having 200 samples per class as set in this paper, meaning there may be fewer samples available. In this section, we conducted experiments on five methods with dataset sizes reduced to 50% and 25% respectively. The experimental accuracies are shown in Tables 15 and 16. Table 15 presents the accuracy of 20-class classification on the USTC-TFC2016 dataset, while Table 16 presents the accuracy of 12-class classification on the ISCX-VPN-NonVPN-2016 dataset. From the tables, we can observe that all five methods exhibit decent stability in this scenario, but the stability of AECCN is slightly higher than the other four methods. When the sample size is reduced to 50% of the original, the accuracy decreased by 1.97% and 3.13% respectively. When the sample size is further reduced to 25%, the accuracy decreased by 7.44% and 7.84% respectively.

**Table 15:** The accuracy of dataset size robustness experiment on USTC-TFC2016 dataset.

	100%	50%	25%
AECCN	98.01 ± 0.1	96.04 ± 0.09	90.57 ± 0.09
SAM	84.39 ± 0.11	78.97 ± 0.14	73.35 ± 0.13
CNN	79.53 ± 0.17	73.44 ± 0.16	68.53 ± 0.15
KNN	63.95 ± 0.16	58.55 ± 0.22	52.16 ± 0.23
ET-BERT	82.02 ± 0.13	79.07 ± 0.1	75.69 ± 0.11
NetMamb	81.57 ± 0.13	73.57 ± 0.13	70.93 ± 0.09

**Table 16:** The accuracy of dataset size robustness experiment on ISCX-VPN-NonVPN-2016 dataset.

	100%	50%	25%
AECCN	88.51 ± 0.12	85.38 ± 0.11	80.67 ± 0.1
SAM	59.95 ± 0.11	54.77 ± 0.12	41.69 ± 0.14
CNN	70.38 ± 0.11	67.51 ± 0.16	57.38 ± 0.12
KNN	54.78 ± 0.19	49.6 ± 0.23	41.61 ± 0.22
ET-BERT	54.59 ± 0.13	49.14 ± 0.11	38.19 ± 0.13
NetMamb	66.15 ± 0.14	55.85 ± 0.15	50.78 ± 0.14

#### 4.3.2. Robustness of noise level.

In the process of collecting raw traffic data, noise may inadvertently be introduced, which is quite common in practical applications. To address this, we introduced Gaussian noise into the original dataset to simulate this scenario and compare the robustness of the five methods under different levels of noise. In this section, we conducted experiments by adding Gaussian noise with mean 0 and variances of 0.2, 0.4, and 0.6 to the original dataset, respectively.

**Fig 10:**
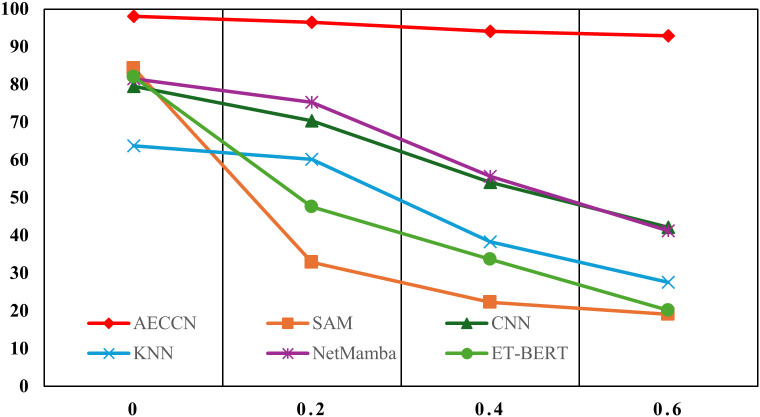
The accuracy of dataset noise level robustness experiment on USTC-TFC2016 dataset.

**Fig 11:**
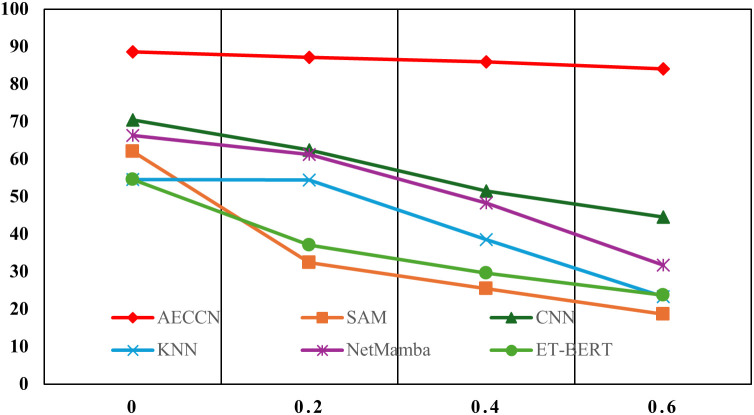
The accuracy of dataset noise level robustness experiment on ISCX-VPN-NonVPN-2016 dataset.

The experimental accuracies are shown in Figs 10 and 11 respectively. Fig 10 displays the accuracy of 20-class classification on the USTC-TFC2016 dataset, while Fig 11 presents the accuracy of 12-class classification on the ISCX-VPN-NonVPN-2016 dataset. From the figures, it is evident that AECCN exhibits greater stability in the presence of noise compared to the other four methods, contrasting with its performance in robustness to dataset sample size. This is mainly attributed to the use of AE for traffic reconstruction in AECCN, as AE is known for its denoising capabilities.

### 4.4. Ablation experiment

To explore the interpretability of AECCN, in this section, we conducted ablation experiments on AECCN to demonstrate the roles and contributions of AE in traffic classification. The results of the ablation experiments are shown in Table 17, where AECCN-AE means we do not use AE for traffic reconstruction. From the results of the ablation experiments, it can be seen that using AE for traffic reconstruction is crucial, which also indicates that zero-padding indeed has a significant impact on classification.

**Table 17:** The accuracy of the ablation experiments on AECCN.

	USTC-TFC2016	MALWARE	NORMAL	ISCX-VPN-NonVPN-2016	VPN	NO-VPN
AECCN-AE	79.53 ± 0.17	88.67 ± 0.14	81.94 ± 0.14	70.38 ± 0.11	92.83 ± 0.15	72.16 ± 0.19
AECCN	98.01 ± 0.1	100	98.73 ± 0.08	88.51 ± 0.12	97.94 ± 0.11	89.6 ± 0.1

## 5. Conclusion

Traffic classification plays a crucial role in various fields such as network management and security. Through effective traffic classification, network administrators can identify and prioritize critical data, ensuring the rational allocation of network resources. Additionally, traffic classification aids in identifying and thwarting potential network attacks, thereby enhancing network security. In this paper, we propose AECCN, a novel traffic classification method. AECCN effectively combines AE and CNN to achieve traffic classification on few-shot datasets. AECCN innovatively leverages the AE to reconstruct traffic data, mitigating the adverse effects of zero-padding, and employs a CNN for classification. The parameter-sharing and local connectivity properties of CNN endow AECCN with strong generalization capabilities, enabling its application to diverse traffic classification scenarios. Experimental results demonstrate its effectiveness in encrypted malicious traffic classification, encrypted normal traffic classification, and VPN-encrypted traffic classification. Compared to existing methods, AECCN achieves a minimum accuracy improvement of 2.86% in six-class VPN-encrypted traffic classification and a maximum gain of 18.13% in six-class normal encrypted traffic classification on the ISCX-VPN-NonVPN-2016 dataset. However, a limitation arises during AE-based reconstruction: shorter traffic instances can only learn feature representations from longer instances within the same class to replace padded zeros. If a traffic class predominantly consists of short sequences, the reconstructed data struggles to learn meaningful representations to substitute zeros. Future work should address this challenge by developing novel methods to enhance feature learning in length-constrained traffic classes.
